# Supplementary material for: Structural basis for reactivating the mutant TERT promoter by cooperative binding of p52 and ETS1
Source: Nat Commun. 2018 Aug 9;9:3183. doi: 10.1038/s41467-018-05644-0 (PMC6085347; doi:10.1038/s41467-018-05644-0)
Supplement: Supplementary file 1 — Supplementary Information [file 41467_2018_5644_MOESM1_ESM.pdf]

Supplementary Information for

Structural Basis for Reactivating the Mutant *TERT* Promoter by cooperative binding of p52 and ETS1

Xueyong Xu[1,#], Yinghui Li[1, #], Sakshibeedu R Bharath[1], Mert Burak Ozturk[1,2], Matthew W. Bowler[3,4], Bryan Zong Lin Loo[1], Vinay Tergaonkar[1,2,5]* and Haiwei Song[1,2]*

[1]Institute of Molecular and Cell Biology, 61 Biopolis Drive, Singapore 138673

[2]Department of Biochemistry, National University of Singapore, 14 Science Drive, Singapore 117543.

[3]European Molecular Biology Laboratory, Grenoble Outstation, 71 avenue des Martyrs, CS 90181 F-38042 Grenoble, France

[4]Unit of Virus Host-Cell Interactions, Univ. Grenoble Alpes-EMBL-CNRS, 71 avenue des Martyrs, CS 90181 F-38042 Grenoble, France

[5]Centre for Cancer Biology, University of South Australia and SA Pathology, Adelaide, South Australia, Australia.

[#]These authors contributed equally to this work

*Correspondence should be addressed to Haiwei Song (haiwei@imcb.a-star.edu.sg) or Vinay Tergaonkar (vinayt@imcb.a-star.edu.sg)

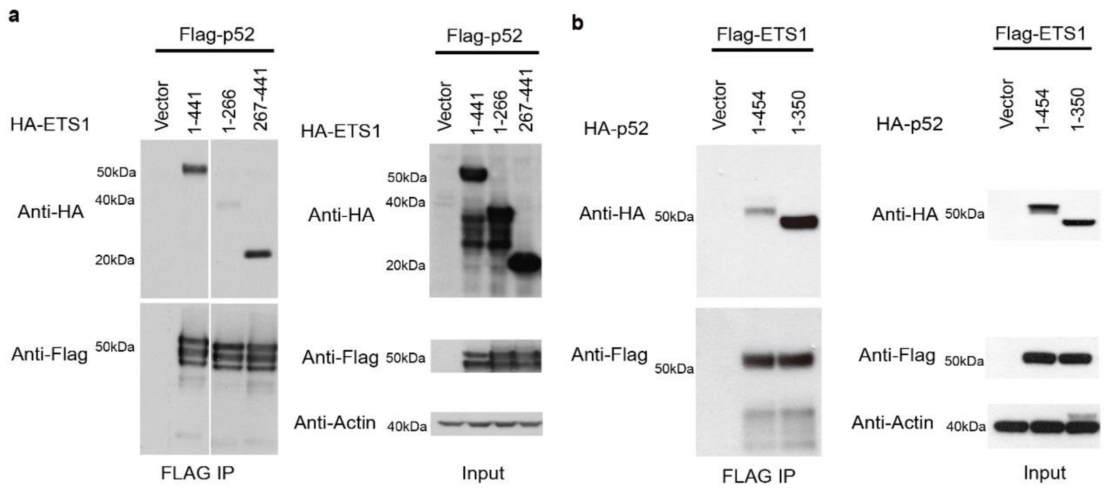

Supplementary Figure 1. Interaction between ETS1 and p52. (**a**) Mapping of the ETS1 regions that interact with p52. Full-length Flag-p52 and HA-ETS1 (full-length and truncations) co-IP with Flag antibody beads followed by western blotting with indicated antibodies. (**b**) Mapping of the p52 regions that interact with ETS1. Full-length Flag-ETS1 and HA-p52 (full-length and truncations) co-IP with Flag antibody beads followed by western blotting with indicated antibodies. Uncropped original scans of all blots are shown in **Supplementary Fig. 11.**

Supplementary Figure 2

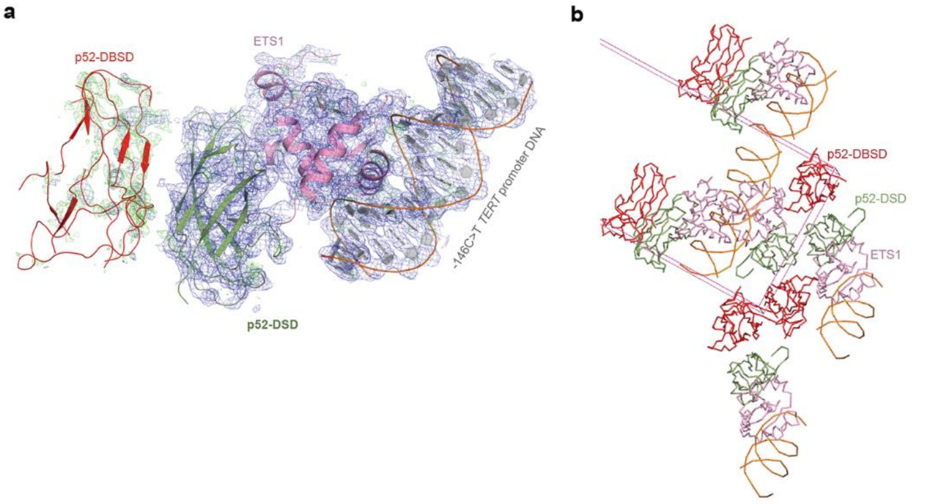

Supplementary Figure 2. The disorder of p52-DBSD in the structure of p52/ETS1/-146C>T complex. (**a**) The 2F_o-F_c and F_o-F_c electron density map are shown as blue mesh contoured at 1.0σ and as green mesh contoured at 3.0σ, respectively. p52-DBSD is roughly fitted into the fragmented electron densities. (**b**) Crystal packing of the p52/ETS1/-146C>T complex.

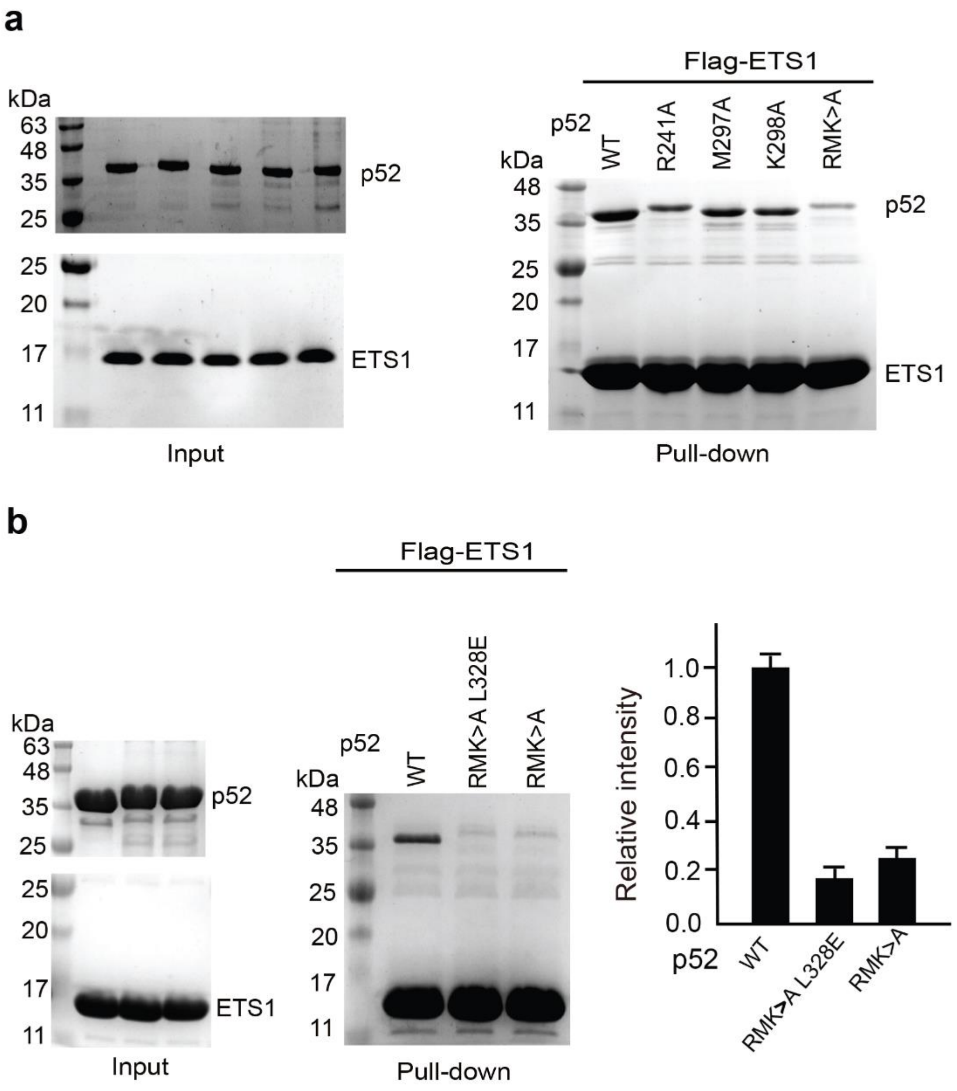

Supplementary Figure 3. Interactions of ETS1 with p52 and its mutants. (**a**) & (**b**) Flag-ETS1$_{331\text{-}441}$ was used to pull-down p52-RHD and its mutants. The bound proteins were analyzed using SDS–PAGE and Coomassie blue staining. The assays were quantified by band densitometry. Error bars, s.d. for triplicate experiments.

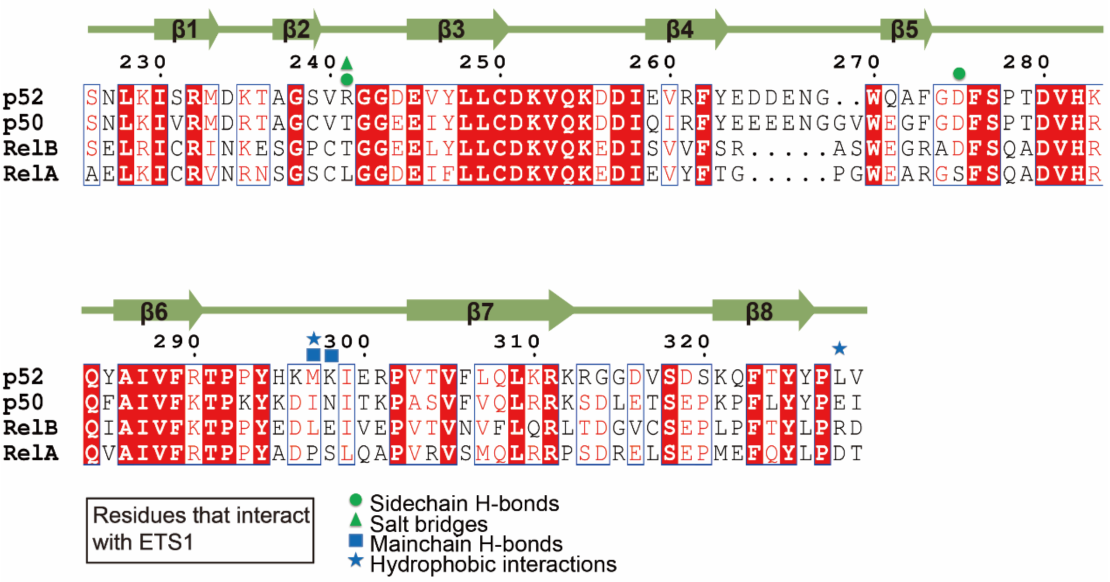

Supplementary Figure 4. Sequence alignment of the dimerization subdomains of NF-κB family members. Identical residues are highlighted with red background, and conserved residues are shown in red characters. Secondary structural elements are indicated and colored in the same color as in Fig. 1a. Residues that form hydrogen bonds with ETS1 via its side-chain and main-chain atoms are indicated as green circles and blue squares, respectively. Residues that form salt bridges are indicated as green triangles. Residues that form hydrophobic interactions are indicated as blue stars.

Supplementary Figure 5

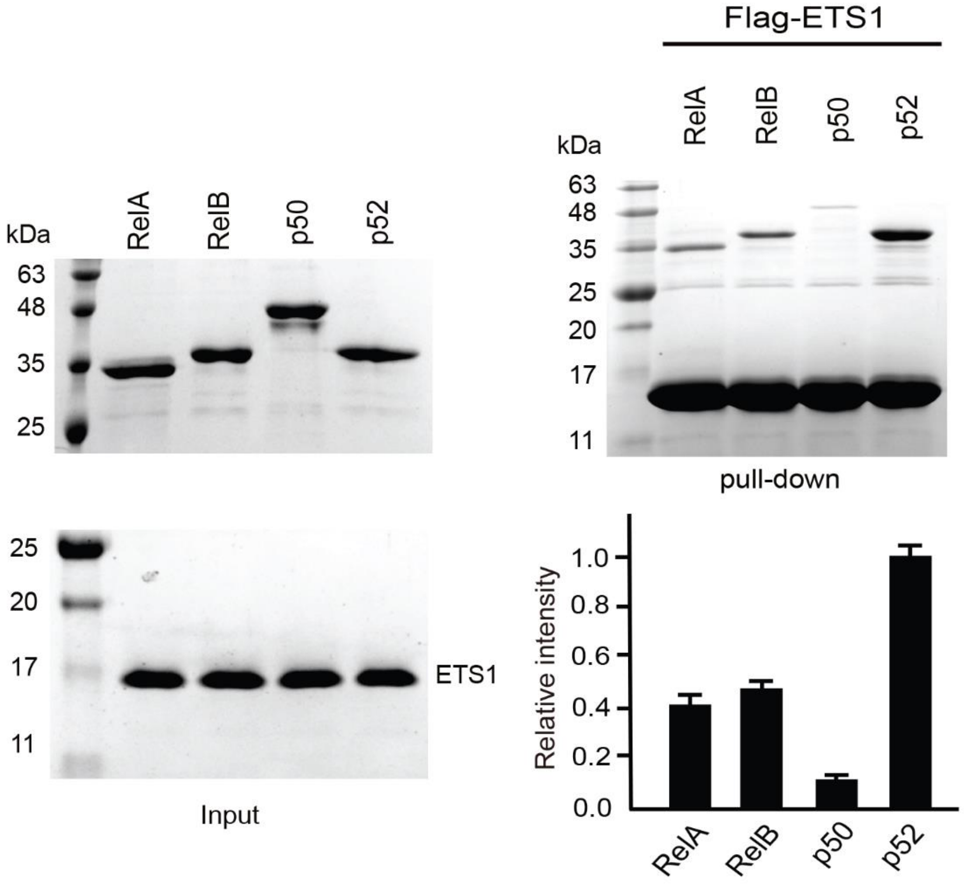

Supplementary Figure 5. Interactions of ETS1 with the NF-κB family members. Flag-ETS1$_{331\text{-}441}$ was used to pull-down the RHD domains of RelA. RelB, p50 and p52. The bound proteins were analyzed using SDS–PAGE and Coomassie blue staining. The assays were quantified by band densitometry. Error bars, s.d. for triplicate experiments.

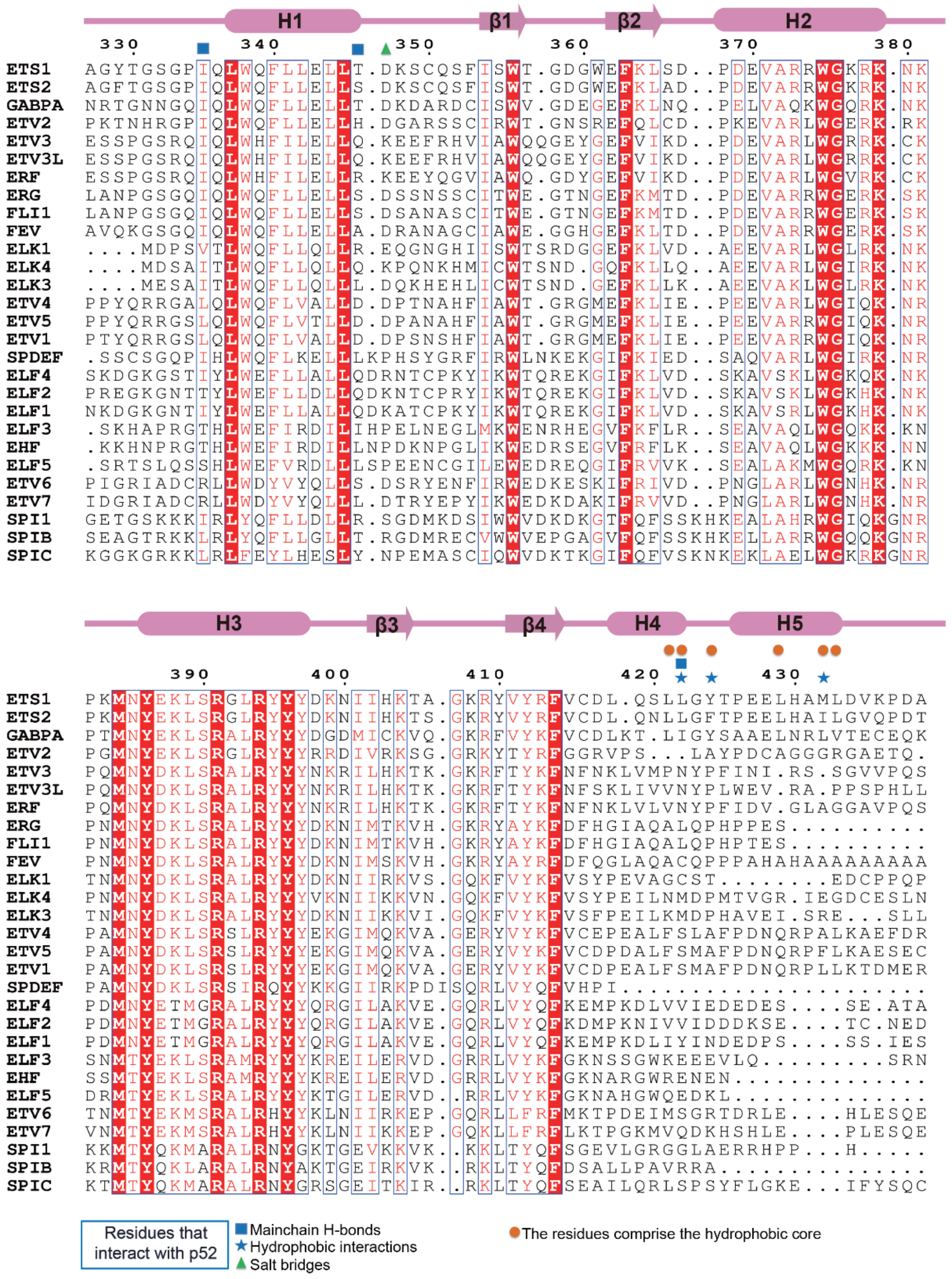

Supplementary Figure 6. Sequence alignment of ETS1$_{297\text{-}439}$ with other ETS family members. Identical residues are highlighted with red background, and conserved residues are shown in red characters. Secondary structural elements are indicated and colored in the same color as in Fig. 1a. Residues that form hydrogen bonds with p52 via main-chain atoms are indicated as blue squares. Residues that form salt bridges are indicated as green triangles. Residues that form hydrophobic interactions are indicated as blue stars. Resides in H4 and H5 that form a hydrophobic core are indicated as orange circles.

Supplementary Figure 7

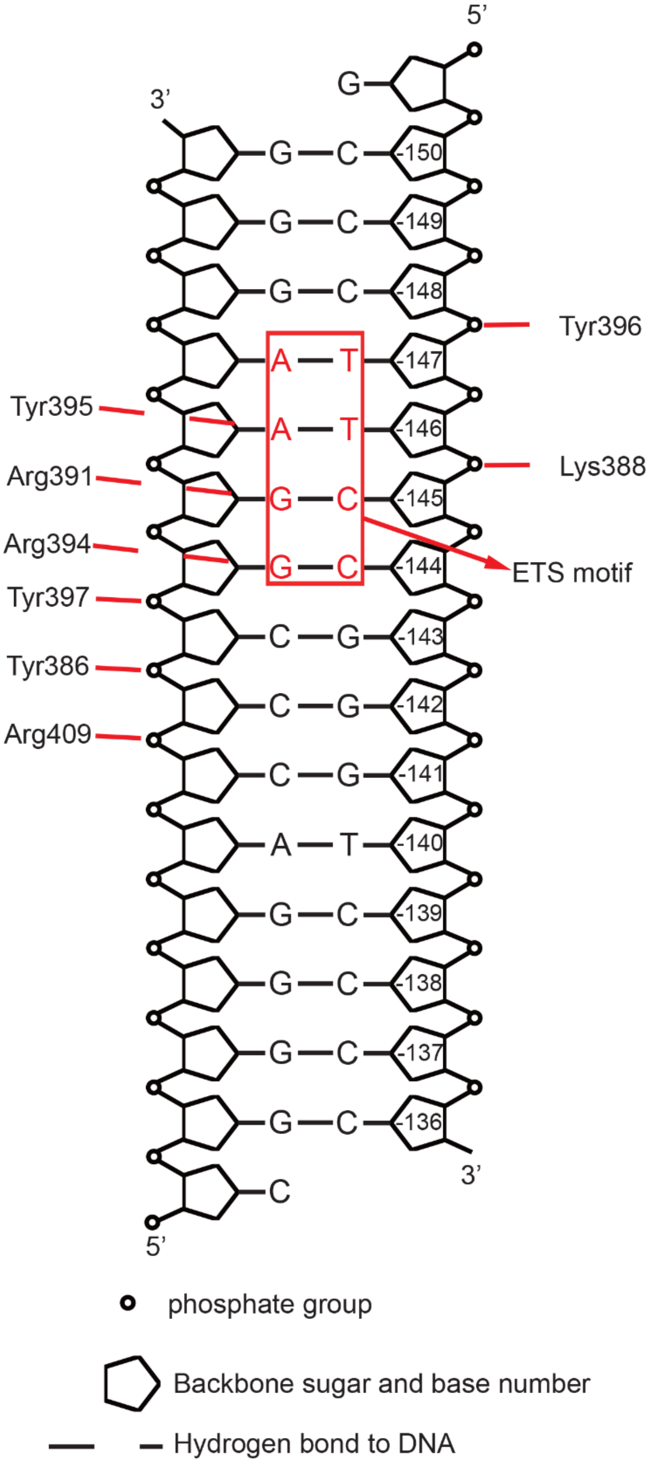

Supplementary Figure 7. Schematic presentation of ETS1-DNA interactions. The hydrogen bonds are indicated as red dashed lines.

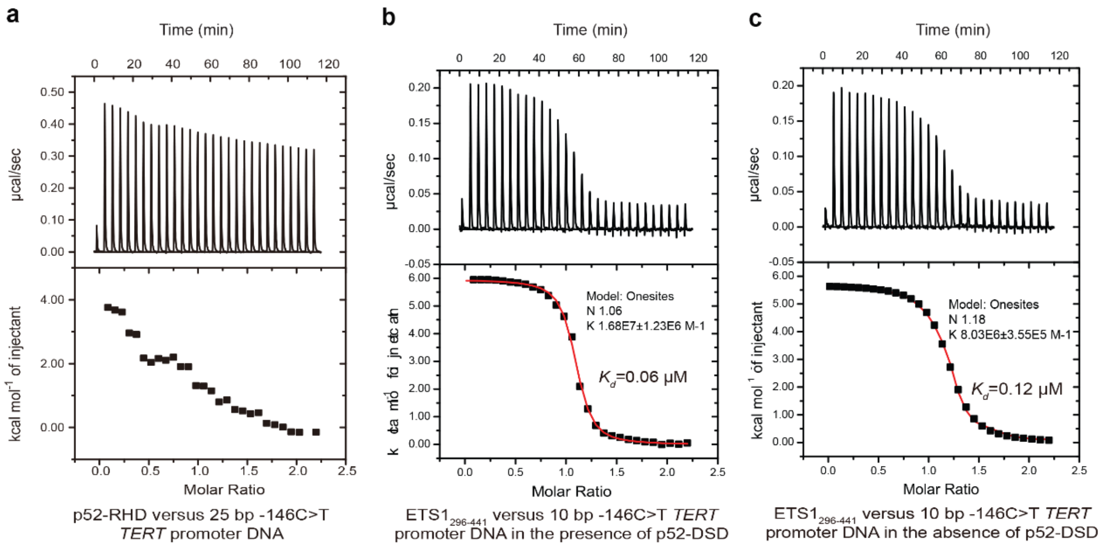

Supplementary Figure 8. ETS1 rather than p52 binds to -146C>T TERT promoter specifically. (**a**) ITC titration of p52-RHD and the 25 bp -146C>T *TERT* promoter DNA. (**b**) ITC titration of ETS1$_{296-441}$ and the 10 bp -146C>T *TERT* promoter DNA in the presence of p52-DSD. (**c**) ITC titration of ETS1$_{296-441}$ and the 10 bp -146C>T *TERT* promoter DNA in the absence of p52-DSD.

Supplementary Figure 9

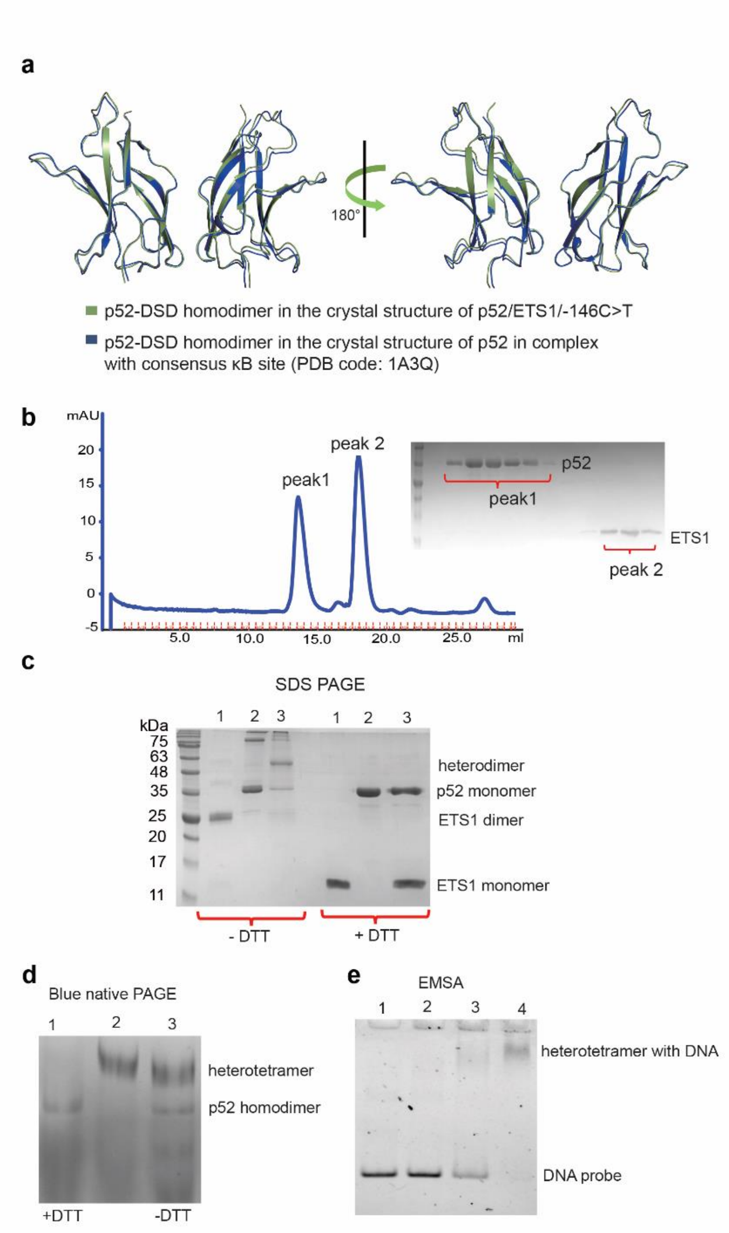

Supplementary Figure 9. p52 and ETS1 can form a p52/ETS1 heterotetramer in solution. (**a**) Superposition of p52-DSD homodimers in the structures of p52/ETS1/-146C>T complex and that in the p52 homodimer-DNA complex (PDB code:1A3Q). (**b**) Gel filtration profile for p52 and ETS1 mixture. (**c**) SDS-PAGE of disulfide crosslinked product. Sample 1: crosslinking of ETS1 alone, Sample 2: crosslinking of p52 alone, Sample 3: crosslinking of p52 and ETS1. (**d**) Blue native PAGE of crosslinked product. Lane 1: p52 and ETS1 crosslinked product with 20 mM DTT, Lane 2: human DNMT$_{646\text{-}1600}$ MW: 108 kDa, Lane 3: p52 and ETS1 crosslinked product without DTT. MW of p52/ETS1 heterotetramer: 94 kDa. (**e**) EMSA of p52/ETS1 heterotetramer with 60 bp -146C>T *TERT* promoter DNA. Lane 1: 1 pmol DNA alone. Lane 2: 1 pmol DNA with 1 pmol heterotetramer, Lane 3: 1 pmol DNA with 3 pmol heterotetramer, Lane 3: 1 pmol DNA together with 6 pmol heterotetramer.

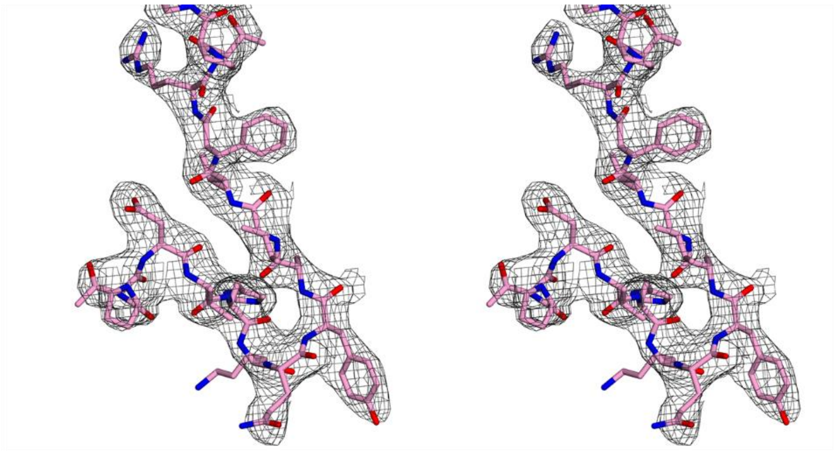

Supplementary Figure 10. Stereo image of a portion of the election density map. The $2F_o$-F_c electron density map is shown as gray mesh contoured at 1.0σ.

Supplementary Figure 11

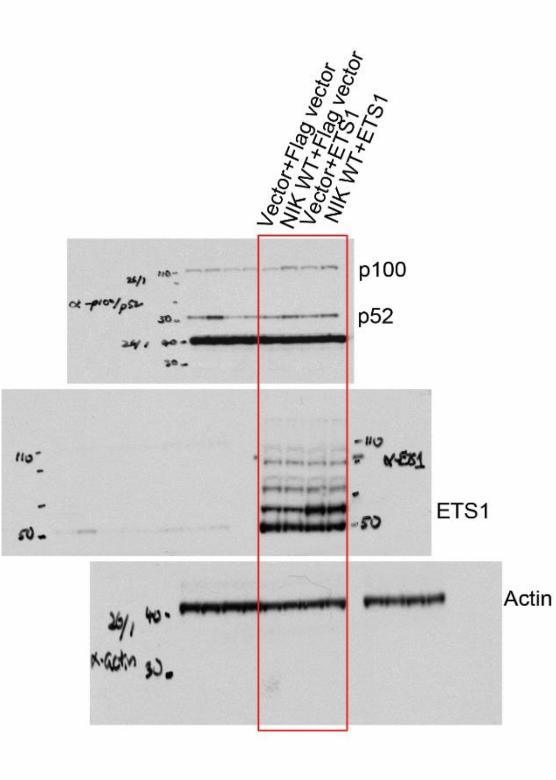

Figure 5e

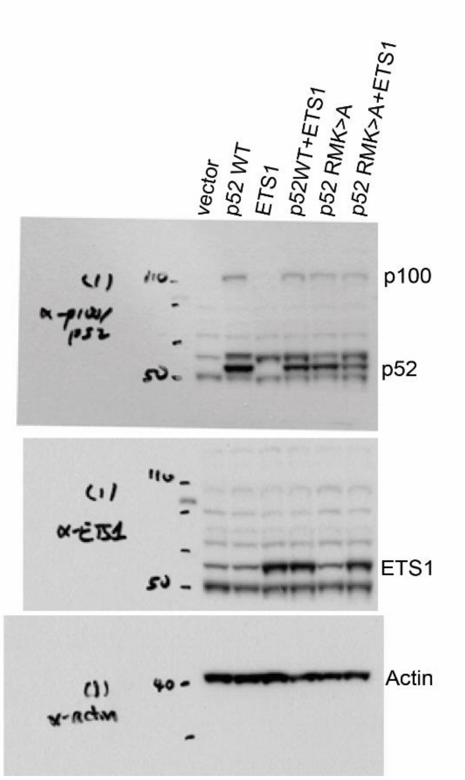

Figure 5f

Supplementary Figure 11 continued

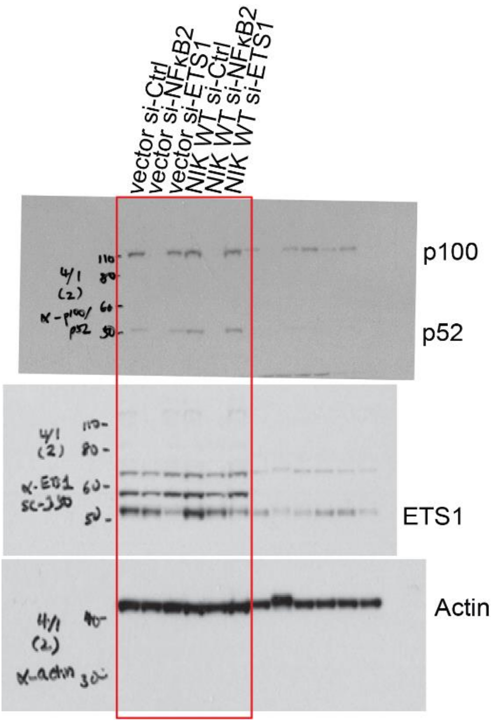

Figure 5g

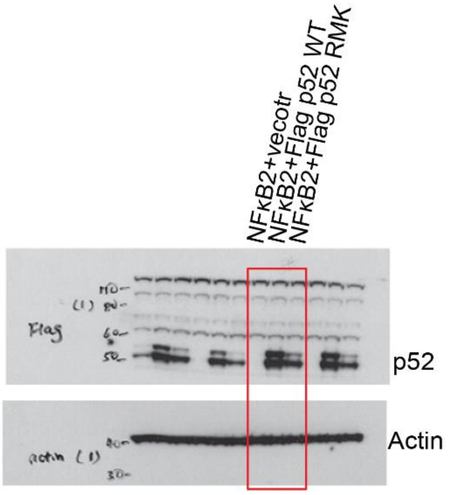

Figure 5h

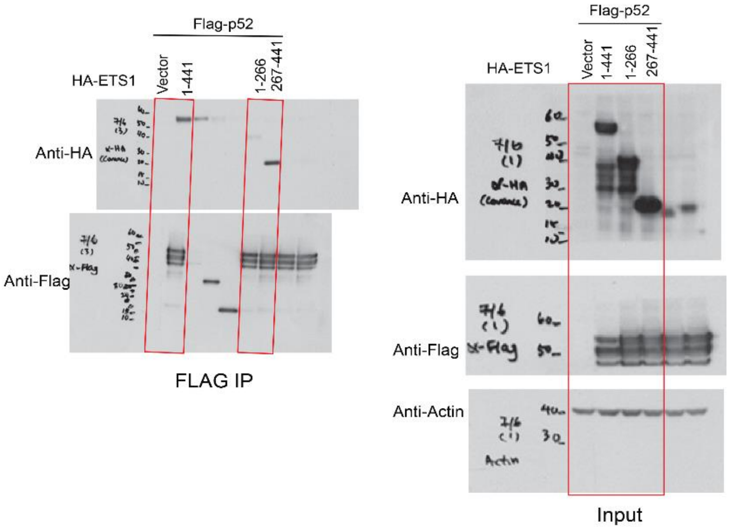

Supplementary Figure 1a

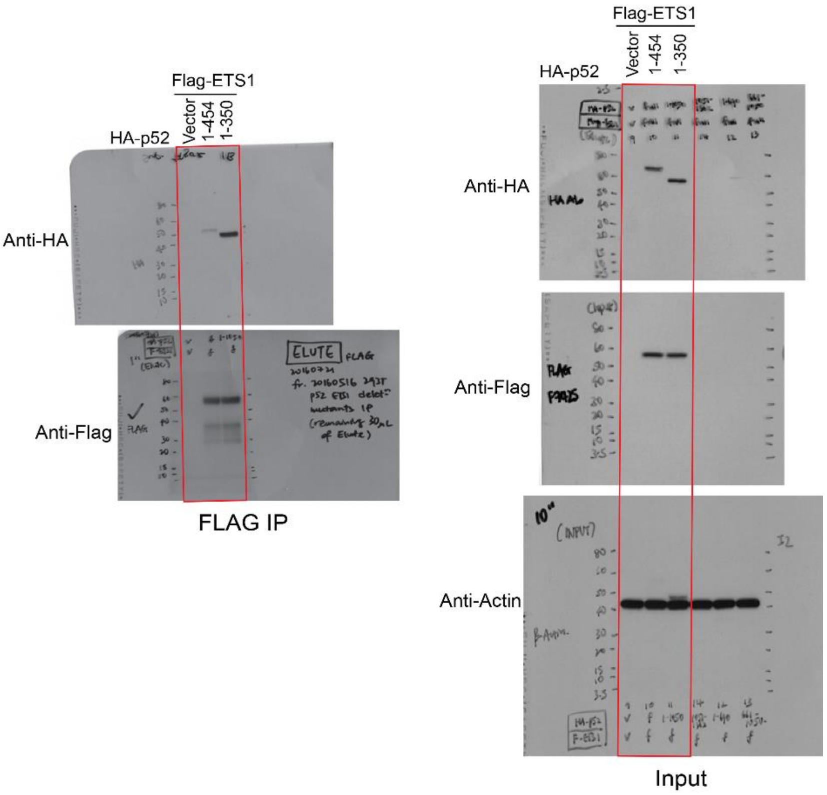

Supplementary Figure 1b

Supplementary Figure 11. Uncropped original western blot scans.

Supplementary Table 1. Data collection and refinement statistics.

	p52/ETS1/-146C>T
**Data Collection**	
Space group	$P\ 4_122$
a, b, c (Å)	71.41, 71.41, 262.52
α, β, γ (°)	90.00, 90.00, 90.00
Resolution (Å)	48.33-2.99 (3.22-2.99)
R_{merge}	0.06 (0.80)
$CC_{1/2}$	0.99 (0.82)
I/sigI	20.3 (2.7)
Completeness	99.9 (100.0)
Redundancy	6.7 (7.2)
**Refinement**	
Resolution (Å)	48.33-2.99 (3.22-2.99)
No. reflections	106712 (18194)
R_{work}/R_{free}	0.26/0.29
R.m.s deviation	
Bond length (Å)	0.004
Bond angle (°)	0.709
No. of atoms	
Protein	1745
dsDNA	656
B-factors	
protein	84.4
dsDNA	76.4
Ramachandran plot statistics (%)	
Favored	95.12
Allowed	4.88
Outliers	0.00

Values in parentheses refer to the highest resolution shell

Supplementary Table 2. Sequence information of DNA used in this work.

**Mutagenesis Primers**	**Forward sequence (5'-> 3')**	**Reverse sequence (5'-> 3')**
p52 (R241A)	cagcaggctctgtggcgggtggagatgaag	cttcatctccacccgccacagagcctgctg
p52 (M297A)	cacccccctatcacaaggcgaagattgagcggcctg	caggccgctcaatcttcgccttgtgatagggggtg
p52 (K298A)	ccccccctatcacaagatggcgattgagcggcctgtaac	gttacaggccgctcaatcgccatcttgtgataggggggg
p52 (M297A and K298A)	cacccccctatcacaaggcggcgattgagcggcctgtaac	gttacaggccgctcaatcgccgccttgtgatagggggggtg
-93T>A	tccccttccttaccgcggccccg	cggggccgcggtaaggaaggggga
-192T>A	gccccttcacctaccagctccgcct	aggcggagctggtaggtgaaggggc
-97C>A	gcccctccccttactttccgcggcc	ggccgcggaaagtaaggggaggggc

**10 bp -146C>T *TERT* promoter DNA for ITC**	**Forward sequence (5'-> 3')**	**Reverse sequence (5'-> 3')**
	cccttccggg	cccggaaggg

**15 bp -146C>T *TERT* promoter DNA for crystal**	**Forward sequence (5'-> 3')**	**Reverse sequence (5'-> 3')**
	gcccttccgggtcccc	cggggacccggaaggg

**25 bp -146C>T *TERT* promoter DNA for ITC**	**Forward sequence (5'-> 3')**	**Reverse sequence (5'-> 3')**
	gtcccgaccccttccgggtccccggc	cgccggggacccggaaggggtcggga

**60 bp -146C>T *TERT* promoter DNA for EMSA***	**Forward sequence (5'-> 3')**	**Reverse sequence (5'-> 3')**
	FAM-cccttccgggtccccggcccagccccctccgggccctcccagcccctccccttcctttcc	ggaaaggaaggggaggggctgggagggcccggagggggctgggccggggacccggaaggg

* DNA fragment from -146C>T to -91 ETS motif of the -146C>T *TERT* promoter
